# Plastic waste generation and emissions from the domestic open burning of plastic waste in Guatemala†

**DOI:** 10.1039/d2ea00082b

**Published:** 2022-11-12

**Authors:** Michelle Bardales Cruz, Eri Saikawa, Mayari Hengstermann, Alexander Ramirez, John P. McCracken, Lisa M. Thompson

**Affiliations:** a Department of Environmental Sciences, Emory University Atlanta GA USA eri.saikawa@emory.edu; b Gangarosa Department of Environmental Health, Emory University Atlanta GA USA; c Universidad del Valle de Guatemala Guatemala City Guatemala; d University of Georgia Athens GA USA; e Nell Hodgson Woodruff School of Nursing, Emory University Atlanta GA USA

## Abstract

Domestic, or household-level, open burning of plastic waste is a source of air pollutants and greenhouse gases that are often neglected in emission inventories. Domestic open burning is a considerable concern in Guatemala due to the lack of access to waste collection services, particularly in rural areas. This paper offers the first attempt to estimate emissions from the domestic open burning of waste at the city and departmental levels in Guatemala. Data were collected from the Xalapán region of Jalapa, Guatemala and analyzed to determine the change in plastic waste generation over time as well as the socioeconomic factors that may affect the extent of plastic waste generation and burning. The annual per capita masses of plastic waste burned were used to estimate emissions from domestic open burning of plastic waste in the region of Xalapán, the cities of Jutiapa and Guatemala city, and all 22 departments in Guatemala. Our results show that rural areas burn more waste domestically, likely because of a lack of access to waste collection, and 30.4% of OC, 24.0% of BC, 23.6% of PM_2.5_, and 2.4% of CO_2_ emissions in Guatemala may not be accounted for by excluding open plastic burning as a source.

## Abstract 2

Domestic, or household-level, open burning of plastic waste is a substantial source of air pollutants and greenhouse gases that are often neglected in Guatemala.

## Graphical abstract

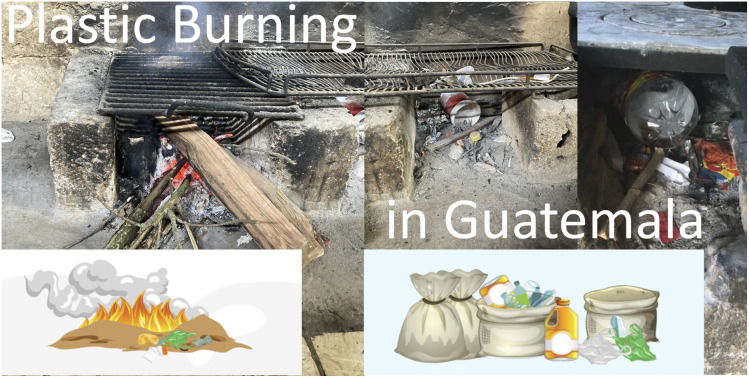

**Environmental significance**

Domestic burning of plastic waste is a source of greenhouse gas (GHG) emissions and household air pollution (HAP) that affects human health. This is of particular concern in countries such as Guatemala, where burning is a main method of waste disposal in rural areas. Current emission inventories do not include emission data on the domestic open burning of plastic waste, which is an under sampled and understudied source of emissions. We find that including emissions from the domestic burning of plastic waste in emission inventories would notably increase current emission estimates. Quantifying such emissions can prevent underestimation of emissions, provide more accurate local, departmental, and national total emission estimates, and inform ways to mitigate the release of GHGs and HAP.

## 1 Introduction

The open burning of plastic waste in domestic, or household-level, fires is a global human health and climate change concern that has not been well studied. Burning of plastic waste is of particular concern in the Global South,^1–4^ especially in countries such as Guatemala, where the management of solid waste has become an issue due to a lack of access to infrastructure, resources, and services necessary to properly manage and dispose of waste.^5^ For this reason, waste is often disposed of through methods that are harmful to the environment, including disposing through burning, burial, or in bodies of water.^6^ This is particularly concerning for plastic waste, as plastics that enter marine ecosystems through these waste disposal methods can accumulate in sediments and induce physiological stress in aquatic organisms and food chains if ingested.^7–10^

In Guatemala, where the present study was conducted, plastics make up approximately 17.3% of waste generated,^11,12^ and 43% of all households dispose of their waste, including plastic, through domestic burning.^6^ These percentages increase in more rural areas of the country, which have fewer waste disposal resources and are in remote locations, far from municipal waste services.^5,6,13^ Residents of such areas must resort to domestic burning to dispose of plastic waste, which poses the risk of releasing toxic air pollutants into their home environment. Thus, waste burning could indicate that waste collection infrastructure must be improved to create better ways to dispose of waste.

Domestic plastic waste burning can occur both in outdoor trash fires and indoor kitchen fires using solid fuel cookstoves. A laboratory study quantifying emissions from cookstoves found that although plastic bags produced fewer emissions than other startup materials, such as kerosene, newspapers, fabric, and wood shims, the burning of plastic bags to ignite fires still contributed to increased cookstove fire emissions.^14^ Plastic waste burning contributes to emissions of greenhouse gases (GHGs) and toxic substances, including carbon dioxide (CO_2_), fine particulate matter with an aerodynamic diameter of 2.5 μm or less (PM_2.5_), black carbon (BC), organic carbon (OC), volatile organic compounds (VOCs), and polycyclic aromatic hydrocarbons (PAHs).^15–19^ The chemical composition of PM_2.5_ due to plastic waste incineration and smoldering fires includes BC, OC, ammonium, chloride, nitrate, sulfate, antimony (Sb), and other trace elements.^16^

Household air pollution (HAP) from the burning of plastic waste in stoves^20^ and cooking fires poses a harmful respiratory, cardiovascular, and overall human health hazard.^21–26^ A recent study measured EFs for solely dry plastic waste burns and found that using dry plastic waste instead of biofuels can decrease open waste burning emissions, although these burns were conducted in improved devices as opposed to those in more traditional stoves.^20^ The health threat from waste burning is still a particular concern for women and children, as they primarily occupy the domestic sphere.^27–29^ In Guatemala, ambient PM_2.5_ exposure is estimated to result in 4105 annual deaths, 2420 total years of healthy life lost due to disability (YLD), and 1.4 billion USD in health damages, including the cost of minimizing the likelihood of premature death, or mortality, and the cost of morbidity based on the country's average wage rates.^30,31^

However, despite being a source of GHG emissions and HAP, domestic waste burning, usually a mix of organic and inorganic materials, like plastic, is scarcely sampled and is understudied as a source of emissions.^17^ Current emission inventories have limited data from domestic burning of plastic waste, which could result in an underestimation of total emissions.^32–34^ Accurately quantifying emissions is important for determining mixing ratios of GHGs and mass concentrations of other air pollutants released into the air from different emission sources.^16,17,28^ Our study examined plastic waste generation in households in one community in the Xalapán region in the Department of Jalapa to estimate emissions of various pollutants from plastic burning and to determine the effects of potential interventions to reduce plastic waste among study participants. In our study, “plastic” refers to mostly single-use household plastic items, such as plastic packaging, bags, plates, utensils, straws, gloves, bottles, and containers, disposed of by the participants in our study.

Since domestic burning of plastic waste is a common form of waste disposal in Guatemala, the purpose of this paper is to estimate emissions and contributions to total national emissions from the domestic burning of plastic waste. This study is the first local and regional emission estimate of chemical species due to the domestic burning of plastic waste in Guatemala. We estimated emissions from domestic plastic waste burning in Guatemala, including: (1) in the La Fuente rural community from the Xalapán region of Jalapa, Guatemala; (2) at the departmental level for Guatemala's 22 departments (Fig. S1†);^6^ (3) in Jutiapa, the capital city of the Department of Jutiapa, which has a population that is about equally urban and rural, and is located approximately 40 kilometers from Jalapa; and (4) in Guatemala city, the capital of the Department of Guatemala, which has a population that is 91% urban.^6^ We initially aimed to investigate the city of Jalapa, which is the city closest to the Xalapán region; however, the World Bank^11,12^ lacked data on the city of Jalapa. Therefore, we chose to estimate emissions in the city of Jutiapa, as it is close in population size and location to Jalapa.

## 2 Methods

### 2.1 Working groups, questionnaire, and data collection

We conducted a 10-week working group session to discuss solid alternatives to burning plastic waste in one rural indigenous community in the Xalapán region of Jalapa, Guatemala. This region lacks access to formal municipal or private waste collection programs. One community was chosen based on accessibility to public transportation, the presence of a large meeting space to conduct weekly activities, and the community leadership committee's approval of the study. All accessible households were visited and asked if they would be interested in participating in any combination of the following three activities: (1) in-home participatory observation of plastic waste disposal; (2) a Knowledge, Attitude, and Practices (KAP) survey;^35^ and (3) a 10-week working group session to discuss alternatives to burning plastic. The working group session was conducted in Spanish and met weekly between June and August 2019. Eighty-seven participants (5 male; 82 female) attended the sessions, which included topics on recycling plastic waste, the adverse effects of plastics on the environment and human health, and how to reduce, reuse, and repurpose plastic waste to improve solid waste management. A sociodemographic questionnaire (see Table S1†) was verbally administered to participants from 50 households who attended the first or second working group sessions. These participants were also provided with burlap bags and asked to collect all plastics that they would have normally burned in an indoor or outdoor fire in the bag for a week. The mass of plastic waste generated per household was weighed, recorded, and taken to the local dump or recycled weekly by our project staff. Each participant collected household plastic waste over a four-week period. The study protocol and informed consent were approved by the Institutional Review Boards (IRB) at the Universidad del Valle de Guatemala and Emory University. Written informed consent was obtained from study participants who participated in the sociodemographic questionnaire and the collection of plastic waste.

### 2.2 Calculating emissions

To quantify emissions from the domestic burning of plastic waste for 62 chemical species in the Xalapán region, we used a Monte Carlo sampling methodology.^36,37^ The emission factors (EFs) that were used to calculate emissions are taken from the Nepal Ambient Monitoring and Source Testing Experiment (NAMaSTE), which took place in 2014, and EF values were measured from burns containing plastics as waste.^16,28^

An EF is a value that contains the unit of mass of a pollutant released into the air per kilogram of the fuel that is burned.^16^ Limited data were available for emission factors (EFs) from waste burning of specifically plastics in traditional stoves. Several studies have measured EFs from waste burns. However, these EFs were either measured from mixed waste burns or fuel-based burns that contained separate, specific kinds of plastic waste (*i.e.*, plastic packaging *versus* plastic foam), rather than exclusively plastic waste burns.^38–41^ The NAMaSTE study is unique in that it measures EF values from homogeneous plastic waste burning that this paper focuses on. Although burning conditions in Nepal and Guatemala would differ, studies measuring EFs from plastic waste burns and conducted in Guatemala were not found. The NAMaSTE study obtained data that most closely fit our purposes of estimating emissions from domestic plastic waste burning fires. We used the NAMaSTE's EF values from fire 16 and plastic burns 1 and 2 (the plastic waste burns)^42,43^ because of the lack of studies measuring EFs for plastic waste burning in Guatemala and NAMaSTE's location in the Global South.

We also quantified the contributions of plastic burning to total emissions, using the data from the Emissions Database for Global Atmospheric Research (EDGAR v5.0), which did not include open domestic burning in their estimates.^16,17,32–34^ First, we created a normal distribution for the mass of plastic waste burned per capita per year, based on the plastic trash data we collected in the field over a month (see Fig. S2†). Second, we created a normal distribution for an EF for each of the 62 chemical species. Then, we conducted one million Monte Carlo samplings, selecting one value for the mass of plastic burned and the other for the EF from the two distributions and multiplied them. This methodology was used to calculate emissions due to the uncertainty associated with the mass and the EF estimates used. The histogram distributions that resulted from running a Monte Carlo simulation indicate the range and probability of possible results for the emission estimates based on this uncertainty. We used the following equation to find the distribution of emissions for each species:*E*_i_ = *M* × EF_i_^44^*E*_i_ – emissions of air pollutant i [g per year]. *M* – mass of plastic burned [kg per year]. EF_i_ – emission factor of air pollutant i [g per kg plastic burned].

To quantify emissions for each of the departments in Guatemala, we first estimated the mass of plastic burned, using the most recent Guatemalan 2018 census data^6^ on population, the number of households, and the number of households that burn waste. We first distributed the average total mass of plastic waste generated annually per capita in Guatemala, using the results from our study (12.2 ± 5.8 kg per capita per year) for a lower boundary estimate and the World Bank value (29.3 kg per capita per year) for an upper boundary estimate.^11,12^ The main cities where data were collected by the World Bank were Guatemala city, Antigua, and Jutiapa, which are higher-income areas located in departments with lower poverty rates than Jalapa, where Xalapán is located (see Table S2†).^6,11,45^ Therefore, lower and upper boundary estimates were used to account for the varying income levels of the locations where data were collected, which could affect the estimated per capita waste generation estimate.^46–52^ To simplify calculations and allow for regression analysis between waste generation per department and socioeconomic and demographic factors in our study, each mass estimate was distributed among the 22 departments in Guatemala according to the population in each department. Individual- or household-level data were not provided by the World Bank to determine the deviation in per capita plastic waste generation. Therefore, the singular value of 29.3 kg per capita per year, rather than a normal distribution, was used for distributing plastic waste generation by the department. We then quantified the mass of plastic burned based on the ratio of households that burn waste in each of the departments, using the census data. Based on this method, the total estimated plastic burned for the population in Guatemala was 80.2 Gg per year for the lower boundary estimate and 193 Gg per year for the upper boundary estimate. Finally, using the estimated mass of plastic burned per department and the EF distribution based on the mean and standard deviation, we created the emission estimates for each department for the 62 chemical species. The estimated per capita emissions were compared among the 22 departments in Guatemala. We also multiplied emissions by the departmental population and summed the results to determine the total annual national emissions from burning plastics for PM_2.5_, BC, OC, CO, and CO_2_.

For the city-level estimates, we used the World Bank data on the mass of total waste generated per capita per day and the percent of total waste that is plastic for Guatemala as a whole, as the World Bank lacked city-specific data for per capita waste generation and plastic waste percent composition. We also used the waste collection rate for Guatemala city (87.5%) and Jutiapa (28.0%), separately.^11,12^ There is no data for Jalapa, so we used the city of Jutiapa as a proxy for Jalapa. We chose Jutiapa because it is the most proximate city (55 km distance) of a similar size to Jalapa, as Jutiapa had a population of 145 880 and Jalapa had a population of 159 840 in 2018.^6^ The difference in waste collection rates in different locations exemplifies the lack of access to waste collection services in more rural areas, such as Jutiapa, as compared to more urban areas like Guatemala city. The rate of waste not collected, provided by the World Bank,^11,12^ was assumed to be burned in order to provide maximum emission estimates from plastic waste burned.

### 2.3 Data analysis

All the data analyses and descriptive statistics, including means, standard deviations, and correlations, as well as significance tests (*i.e.*, *t*-test and ANOVA) were conducted using R version 4.0.2.^53^ Significance tests were used to determine whether education and wealth, measured by cell phone, radio, and color television ownership and internet access, significantly affected the amount of waste generated by the participants. The categories for education were as follows: no formal education, incomplete primary education, complete primary education, and secondary education. The categories for cell phone, radio, and color television ownership and internet access were binary for whether the participants did or did not have ownership or access to these commodities. Monte Carlo sampling and emission calculations were also conducted using R.

## 3 Results and discussion

### 3.1 Demographic information

All respondents to the Xalapán region survey were females, with an average age of 36 ± 11 years, and the median number of people living in each participant's household was six people (see Table 1). Only one participant was studying and had completed 13 years of formal education at the time of our study. The highest level of education completed by most of the participants was incomplete primary education (46%), demonstrating low levels of education received by most of these rural female participants. Most (88%) of the participants did not work outside of the home. The most common stove used for cooking in Jalapa was a poyetón (52%), which is an elevated open fire stove without a chimney and, in terms of pollution, is similar to an open fire stove. Women were in charge of cooking and waste disposal, which included domestic burning of plastic waste or other materials. As a result, they are more prone to harmful air pollutant exposure. Decreasing plastic waste from household burning and cooking could thus reduce exposure to pollution and adverse health-related effects of this exposure.

**Table 1:** Demographic information for the participants in the survey conducted in the Xalapán region of Jalapa, Guatemala (*n* = 50)

Characteristics	Response	
Female (%)	100	
Average age, in years (SD)	36 (11)	
Median number of people living in each household	6	
Stove type used most of the time for cooking at home (%)	Open fire, three stone fire	4
	Poyetón	52
	Improved stove with chimney	38
	Gas	6
	Electric	0
Methods used to dispose of waste in the home (%)	Burning	84
	Buried	22
	Other	4
Participants currently studying (%)		2
Years of formal education of those currently studying		13
Highest level of education completed (%)	Without formal education	22
	Primary, incomplete	46
	Primary, complete	26
	Secondary, incomplete	0
	Secondary, complete	6
	Vocational or technical school	0
	University	0
Occupations of participants (%)	Does not work	52
	Homemaker	36
	Cook	4
	Merchant	2
	Field work	4
	Selling food	2
Cell phone ownership (%)	No cell phone	60
	Own a cell phone (not smartphone)	34
	Own a cell phone (smartphone)	6
Color television ownership (%)		24
Radio ownership (%)		28
Computer ownership (%)		0
Access to the internet (%)		4
Average weight in kg of plastic waste generated in the 1st week (SD)		1.31 (0.66)
Average weight in kg of plastic waste generated in the 2nd week (SD)		1.12 (0.91)
Average weight in kg of plastic waste generated in the 3rd week (SD)		0.92 (0.70)
Average weight in kg of plastic waste generated in the 4th week (SD)		0.99 (0.77)

### 3.2 Plastic waste generation in the Xalapán region of Jalapa, Guatemala

The average weight of plastic waste generated per household for weeks 1 through 4 in the study was 1.31 ± 0.66 kg, 1.12 ± 0.91 kg, 0.92 ± 0.70 kg, and 0.99 ± 0.77 kg, respectively. The average amount of plastic waste generated by the participants in Xalapán over the four-week period was 3.34 × 10^−2^ ± 1.58 × 10^−2^ kg per person per day (see Table S3†).

We conducted hypothesis tests to determine: (1) whether the average amount of plastic waste generated per household significantly changed during the four weeks; and (2) whether ownership of assets (cell phone, radio, color television, computer, internet access) and level of education had a statistically significant impact on the mass of domestic plastic waste generated. Since the distribution of the total mass of plastic waste generated per household was slightly right skewed, the variable for the mass of plastic waste generated per household was log-transformed when conducting the significance tests.

**Fig. 1:**
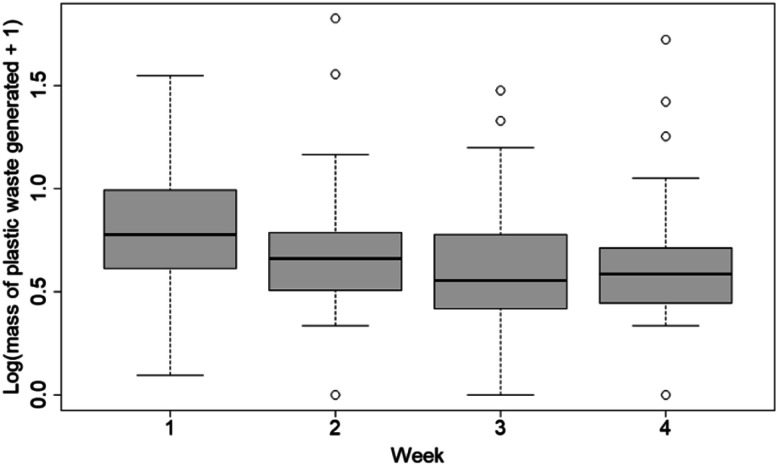
Boxplot of plastic waste in kilograms generated by each household over the four weeks of the survey. The average mass of plastic waste generated in the participating households is represented by the blue points on the boxplot.

There was an overall decrease in the average amount of plastic waste generated per household over time (Fig. 1). The decrease in plastic generation was significant when comparing the plastic waste generation of week 1 to week 2 (two-tailed two-sample *t*-test, *p* = 0.07), week 3 (two-tailed two-sample *t*-test, *p* = 0.001), and to week 4 (two-tailed two-sample *t*-test, *p* = 0.007; see Table S4†). These results suggest that the workshops and trainings conducted during the study could have contributed to a reduction in the amount of plastic waste generated throughout the course of the study. There was a very slight increase in the mass of waste generated from week 3 to week 4; however, this difference was insignificant (two-tailed two-sample *t*-test, *p* = 0.51). This result could be a preliminary indication of a plateau point for the extent of the effect of the workshops on the average mass of plastic waste generated. However, the participants did see this study as an opportunity to dispose of items that are not easily disposable and that they had not been able to dispose of due to a lack of access to formal waste collection. So the first measurements were more likely to contain more larger plastic items, such as broken plastic chairs or toys. Therefore, the decrease in the mass of plastic waste generated over the four weeks could be affected by a decrease in large-mass plastic items being brought for measurement and disposal. There was not a significant correlation between the number of persons living in a household and the weight of plastic waste generated (*r* = −0.08, *p* = 0.58). This result suggests that other factors may better explain the difference in the amount of waste produced by each household.

As shown in Table 2, no significant relationship was observed between the amount of plastic waste generated and education level (*F* = 0.35). Those who owned a cell phone generated more waste overall, but the difference in waste generation between cell phone owners and non-owners was not significant (two-tailed two-sample *t*-test, *p* = 0.62). Those who owned a radio generated more waste overall, but the difference between radio ownership and non-ownership was insignificant as well (two-tailed two-sample *t*-test, *p* = 0.50). The relationships between the amount of plastic generated and: (1) color television ownership (two-tailed two-sample *t*-test, *p* = 0.05) and (2) internet access (two-tailed two-sample *t*-test, *p* = 0.07) were found to be statistically significant. Those with a color television or internet access generated significantly less plastic waste than those without a color television or without internet access. This relationship between television or internet access and the mass of plastic waste generated may result from both variables being indicative of higher income households that are more likely to afford durable items. The purchase and use of cheaper, disposable, and single-use plastic items is more likely in lower income households that cannot afford higher quality items. These differences would thus contribute to higher amounts of plastic waste being produced in lower income households. Our results can be compared to a previous study that found that non-biodegradable, including plastic, waste generation decreases from low income to lower middle-income groups, although this waste generation then increases for middle, upper-middle, and highest income groups.^54^ Other studies have found organic or total solid waste generation to be positively correlated with the household income level,^46,47,49–52^ suggesting that waste composition and the amount of waste produced per waste type depend on the income level.

**Table 2:** Significance test results for the relationship between the mass of waste generated and education level; cellphone, radio and color television ownership; and internet access

Testing for relationship between the mass of waste generated	Difference in average plastic waste generation (kg)	Test	*p*-Value
Education level^a^	0.715	ANOVA	0.32
Cell phone ownership	−0.26	*t*-Test	0.79
Radio ownership	0.440	*t*-Test	0.51
Color television ownership	−1.21	*t*-Test	0.05
Internet access	−0.902	*t*-Test	0.07

a Average absolute value difference in plastic waste generation among 4 categories of education (no formal education, incomplete primary education, complete primary education, and secondary education).

Income data are needed to determine the factors that are indicators of wealth in the rural community of Xalapán, Guatemala, so we can test whether the correlation found with color television and internet access is related to wealth. In this study we chose not to ask working group participants about their household income levels as this would have been perceived as invasive.

### 3.3 Plastic waste burning emission estimates

**Fig. 2:**
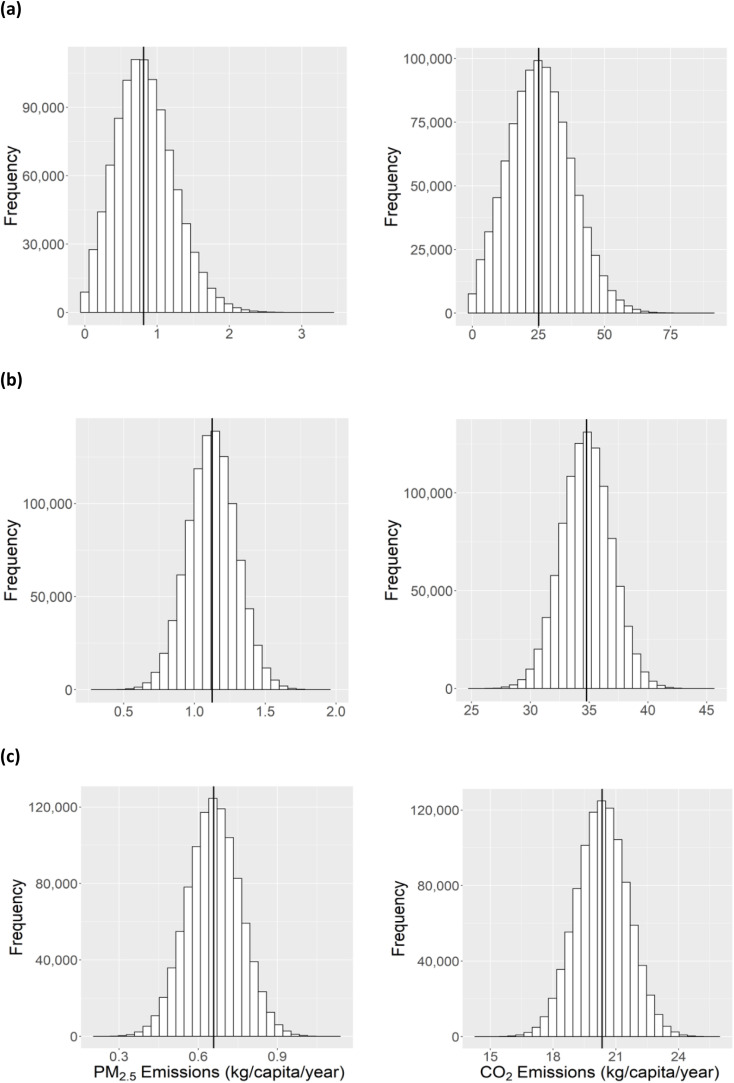
Particulate matter (PM_2.5_, left) and carbon dioxide (CO_2_, right) annual emission estimate distribution from Monte Carlo samplings for (a) the Xalapán region of Jalapa, (b) the city of Jutiapa, and (c) Guatemala city.

The estimated emissions for PM_2.5_ and CO_2_ in the Xalapán region are shown in Fig. 2. Based on the Xalapán survey, 84% of households indicated that they burned their waste as their primary mode of waste disposal (see Table S3†). Among the people whose primary mode of disposal is burning, the quantified average amount of plastic waste that would have been burned per person per day was 2.66 × 10^−2^ ± 1.32 × 10^−2^ kg (see Table S3†) based on the waste collected. Comparing this with the total mass of plastic waste collected, it was found that approximately 80% of the mass of plastic waste generated came from households that use waste burning as their primary mode of waste disposal (see Table S3†). This percentage (80%) compares well with the percentage of households that burn waste as their primary mode of waste disposal in Xalapán (84%). These results indicate that the percentage of households that primarily disposes of waste through burning could be used as a proxy for calculating the mass of plastic waste burned. We thus used this value to estimate the average mass of plastic burned in each department in Guatemala.

**Fig. 3:**
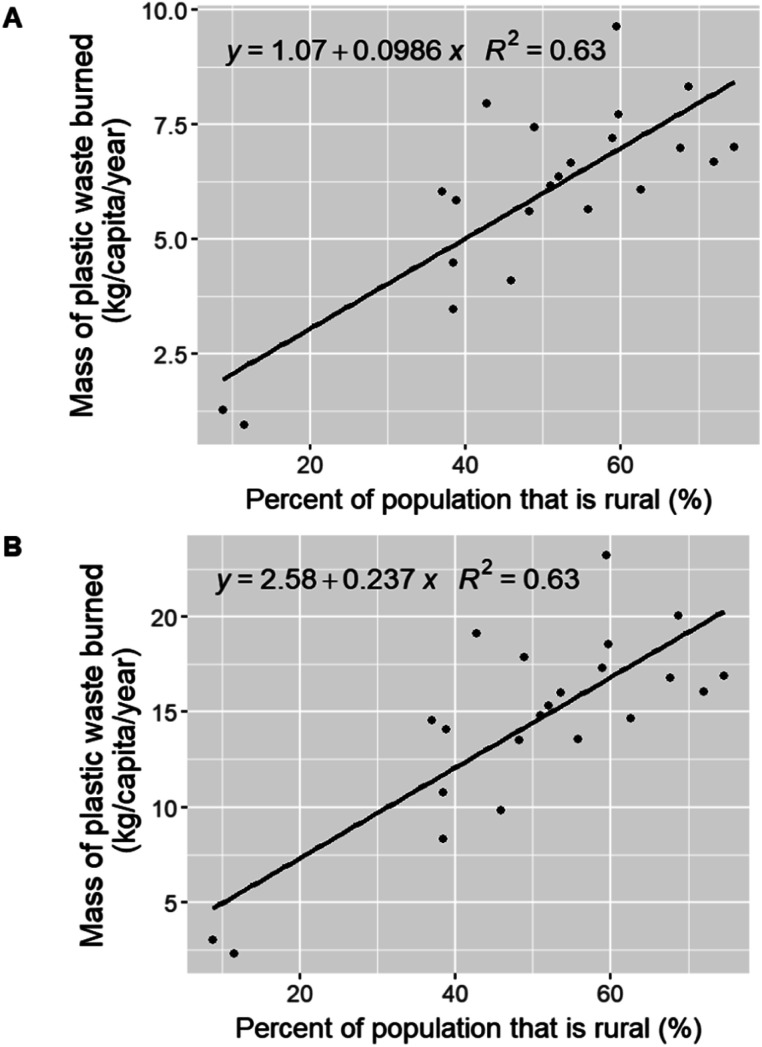
Scatterplot of the percent of the population that is rural in each Guatemalan department *versus* the average mass of plastic waste burned annually per capita in each department using the lower (A) and upper (B) boundary estimates.

The lower and upper boundary estimated mass of plastic waste burned at the department level in Guatemala based on the 2018 Guatemalan census data are shown in Table S2.† There is a large range from 0.645 kg per person per year to 61.4 kg per person per year, depending on the location. One reason for this range is likely the difference in access to private or municipal waste collection services in urban *versus* rural areas within each department. There is a moderate positive correlation between the percentage of the population in each department that is rural, and the average mass of plastic waste burned per capita per year (Fig. 3). A simple linear regression model between these variables has an *R*^2^ value of about 0.63, which suggests that about 63% of the variance in the average mass of plastic waste burned domestically can be explained by the size of the rural population.

**Fig. 4:**
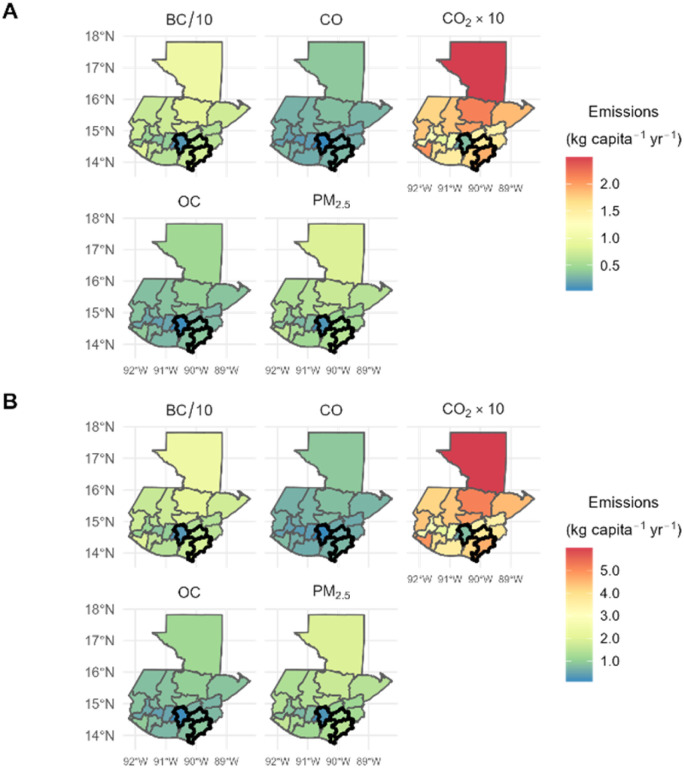
Per capita emission estimates of black carbon (BC), methane (CH_4_), carbon monoxide (CO), carbon dioxide (CO_2_), organic carbon (OC), and fine particulate matter (PM_2.5_) from burning plastics in each Department in Guatemala. Emission estimates indicated by corresponding colors on the legend are divided by 10 for BC (BC/10), and multiplied by 10 for CO_2_ (CO_2_ × 10) due to differences in magnitude. The borders of the departments of Guatemala (left), Jalapa (upper right), and Jutiapa (bottom right) are bolded in black.

The estimated average mass of plastic waste burned based on the Guatemalan census data was higher in the Department of Jutiapa (7.44 ± 3.52 kg per capita per year lower bound estimate; 17.9 kg per capita per year upper bound estimate) than in the Department of Jalapa (6.04 ± 2.86 kg per capita per year lower bound estimate; 14.5 kg per capita per year upper bound estimate) and the Department of Guatemala (1.26 ± 0.594 kg per capita per year lower bound estimate; 3.02 kg per capita per year upper bound estimate). The mass of plastic waste generated in the cities of Guatemala city and Jutiapa was 28.1 kg per capita per year and 15.4 kg per capita per year, respectively (see Table S5†). The percent of plastic waste assumed to be burned in Jutiapa (87.5%) was notably higher than that of Guatemala city (28.0%). Although the per capita plastic waste generation in Guatemala city is about two times that of Jutiapa, a lower mass of plastic waste is domestically burned in Guatemala city (7.87 kg per year), as compared to Jutiapa (13.5 kg per year). This difference in the waste collection rate, and thus assumed domestic waste burning rate, highlights the lack of access to waste collection services in more rural areas, even cities with a population over 100 000, such as Jutiapa and Jalapa, compared to Guatemala city. Using this estimated mass of plastic waste burned, we calculated emission estimates for the 62 species at the department level in Guatemala using both methods (see Tables S6 and S7†). Maps of the distribution of emissions of six pollutants in 22 departments are shown in Fig. 4.

EDGAR v5.0 currently does not include emissions from the domestic burning of plastic waste. Our results show that including plastic waste burning in the EDGAR v5.0 emissions inventory could increase national emission estimates by between 6.71 × 10^6^ ± 3.37 × 10^6^ kg per year (5.62 ± 2.82%; lower boundary estimate) and 1.61 × 10^7^ ± 2.46 × 10^6^ kg per year (13.5 ± 2.06%; upper boundary estimate) for PM_2.5_ and by between 2.07 × 10^8^ ± 9.90 × 10^7^ kg per year (0.564 ± 0.369%; lower boundary estimate) and (4.99 × 10^8^ ± 3.03 × 10^7^) (1.36 ± 8.25 × 10^−2^%; upper boundary estimate) for CO_2_ (see Table 3). Table 3 also summarizes the estimated total emissions and percent increases in national emissions for BC, OC, and CO emissions, if domestic burning of plastic waste was included in the EDGAR v5.0 (ref. 32–34) emission inventory.

**Table 3:** Lower and upper boundary city-level, departmental, and national emission estimates from plastic waste burning for PM_2.5_, EC, OC, CO, and CO_2_

Data source	Value (units)	Location	Species				
			PM_2.5_	BC	OC	CO	CO_2_
World Bank: city level	Plastic waste burning emissions (kg per capita per year)	Xalapán, Jalapa (SD)	0.813 (±0.425)	0.100 (±5.08 × 10^−2^)	0.485 (±0.250)	0.384 (±0.398)	25.1 (±12.5)
		City of Jutiapa (SD)	1.13 (±0.171)	0.139 (±1.40 × 10^−2^)	0.671 (±8.88 × 10^−2^)	0.531 (±0.434)	34.8 (±2.11)
		Guatemala city (SD)	0.659 (±0.100)	8.12 × 10^−2^ (±8.17 × 10^−3^)	0.393 (±5.20 × 10^−2^)	0.310 (±0.254)	20.3 (±1.24)
La Fuente, Jalapa study	Plastic waste burning emissions – lower boundary estimates (kg per capita per year)	Department of Jalapa (SD)	0.505 (±0.254)	6.23 × 10^−2^ (±3.03 × 10^−2^)	0.301 (±0.149)	0.238 (±0.243)	15.6 (±0.747)
		Department of Jutiapa (SD)	0.623 (±0.313)	7.67 × 10^−2^ (±3.73 × 10^−2^)	0.371 (±0.184)	0.293 (±0.300)	19.2 (±9.19)
		Department of Guatemala (SD)	0.105 (±5.25 × 10^−2^)	1.30 × 10^−2^ (±6.26 × 10^−3^)	6.28 × 10^−2^ (±3.09 × 10^−2^)	4.96 × 10^−2^ (±5.06 × 10^−2^)	3.25 (±1.54)
	Total plastic waste burning emissions – lower boundary estimate (kg per year)	All departments	6.71 × 10^6^ (±3.37 × 10^6^)	8.27 × 10^5^ (±4.02 × 10^5^)	4.00 × 10^6^ (±1.98 × 10^6^)	3.16 × 10^6^ (±3.23 × 10^6^)	2.07 × 10^8^ (±9.90 × 10^7^)
Guatemala census: department level	Plastic waste burning emissions – upper boundary estimate (kg per capita per year)	Department of Jalapa (SD)	1.21 (±0.184)	0.150 (±1.51 × 10^−2^)	0.723 (±9.58 × 10^−2^)	0.571 (±0.468)	37.5 (±2.28)
		Department of Jutiapa (SD)	1.50 (±0.227)	0.185 (±1.86 × 10^−2^)	0.893 (±0.118)	0.705 (±0.577)	46.3 (±2.81)
		Department of Guatemala (SD)	0.253 (±3.85 × 10^−2^)	3.11 × 10^−2^ (±3.14 × 10^−3^)	0.151 (±2.00 × 10^−2^)	0.119 (±9.73 × 10^−2^)	7.81 (±0.474)
	Total plastic waste burning emissions – upper boundary estimate (kg per year)	All departments	1.61 × 10^7^ (±2.46 × 10^6^)	1.99 × 10^6^ (±2.01 × 10^5^)	9.62 × 10^6^ (±1.28 × 10^6^)	7.61 × 10^6^ (±6.21 × 10^6^)	4.99 × 10^8^ (±3.03 × 10^7^)
EDGAR v5.0	Total emissions (kg in 2015)	Guatemala	1.19 × 10^8^	1.45 × 10^7^	5.54 × 10^7^	2.05 × 10^9^	3.68 × 10^10^
	Percent increase in national emissions – lower boundary estimate (%)	Guatemala	5.62 (±2.82)	5.70 (±2.77)	7.23 (±3.58)	0.154 (±0.157)	0.564 (±0.369)
	Percent increase in national emissions – upper boundary estimate (%)	Guatemala	13.5 (±2.06)	13.7 (±1.38)	17.4 (±2.30)	0.371 (±0.303)	1.36 (±8.25 × 10^−2^)
	Difference in lower and upper boundary percent increase estimates (%)	Guatemala	7.90 (±0.762)	8.01 (±1.39)	10.2 (±1.27)	0.217 (±0.146)	0.792 (±0.187)

Estimated emissions from the domestic burning of plastic waste ranged from 2.63 × 10^4^ ± 1.32 × 10^4^ kg per year for PM_2.5_ and 8.11 × 10^5^ ± 3.87 × 10^5^ kg per year for CO_2_ in the Department of Sacatepéquez to 8.46 × 10^5^ ± 4.25 × 10^5^ kg per year for PM_2.5_ and 2.61 × 10^7^ ± 1.25 × 10^7^ kg per year for CO_2_ in the Department of Alta Verapaz for the lower boundary estimate (see Table S8†). For the upper boundary estimate, they ranged from 6.33 × 10^4^ ± 9.64 × 10^3^ kg per year for PM_2.5_ and 1.96 × 10^6^ ± 1.19 × 10^5^ kg per year for CO_2_ in the Department of Sacatepéquez to 2.03 × 10^6^ ± 3.10 × 10^5^ kg per year for PM_2.5_ and 6.28 × 10^7^ ± 3.82 × 10^6^ kg per year for CO_2_ in the Department of Alta Verapaz (see Table S9†). Most of the population in Alta Verapaz (68.7%) lives in rural areas (see Table S2†), which may contribute to a lack of access to nearby waste disposal services, leading to high emissions from plastic burning in the department. According to the Instituto Nacional de Estadística Guatemala (INE; National Statistics Institute of Guatemala),^55^ the Department of Alta Verapaz had the highest poverty rate in the country in 2014, with 83.1% of its population experiencing poverty (see Table S2†). The socioeconomic status of the Department of Alta Verapaz may contribute to a lack of monetary resources for establishing formal waste disposal programs. The Department of Alta Verapaz, similar to Xalapán, also has a high indigenous population, with 93.2% of its population being indigenous (see Table S2†). Therefore, a similar reduction strategy might be helpful for the regions of Xalapán and Alta Verapaz. Providing access to information and education about the benefits of waste reduction and the risks of waste burning to human health and the environment were possible strategies in Xalapán that could be useful in Alta Verapaz. More importantly, however, increasing financial resources for waste disposal and establishing local waste disposal infrastructure would help decrease waste burning as a method of disposal and reduce GHG and air pollutant emissions in these areas. The information presented in this study may be useful in determining the extent and types of interventions that should take place to improve waste collection infrastructure and thus reduce plastic waste generation in other similar communities.

## 4 Conclusions

Our results show that including emissions from the domestic burning of plastic waste in emission inventories could result in notable increases in emission estimates, including 7.23 ± 3.58 to 17.4 ± 2.3% for OC, 5.70 ± 2.77 to 13.7 ± 1.38% for BC, 5.62 ± 2.82 to 13.5 ± 2.06% for PM_2.5_, and 0.564 ± 0.369 to 1.36 ± 8.25 × 10^−2^% for CO_2_, and 0.154 ± 0.157 to 0.371 ± 0.303% for CO (see Table 3). Furthermore, providing access to proper waste disposal programs in low-resource countries, especially in rural areas, could mitigate the amount of waste burned domestically and thus reduce indoor and outdoor air pollution from this source.

Limitations of this study include a small sample size and a short length of the data collection period for the mass of waste generated by the participants in the Xalapán survey. A larger sample of households and a longer period of data collection would provide a more reliable estimate of the average mass of plastic waste generated and burned. Data on the mass of plastic waste generated by each household before the workshops were also not collected and introduced a limitation for estimating emissions. More data are needed regarding emissions from domestic burning of plastic waste both before and after any waste reduction intervention from the workshops to provide a baseline for comparison and to assess the efficacy of the intervention. Future studies may also improve upon this study's data collection by gathering information on corresponding household income levels and fuel types used in order to determine whether or how these factors would affect plastic waste generation and emissions released when burning.

Another limitation is that the EFs used to calculate emissions in our study were not local to Guatemala due to a lack of studies quantifying EFs from homogeneous plastic waste burning. More data on EFs from plastic waste burning in Guatemala are needed to provide more accurate local estimates. Collecting data on the division of the mass of plastic waste burned outdoors *versus* indoors would also help address this uncertainty. The current lack of data on the mass of plastic waste disposed of through domestic burning is also a limitation our study faced. Our emission estimates at the city level relied on the assumption that any waste that was not collected by private or municipal waste collection service was burned, even though other forms of plastic waste disposal, such as burial or dumping in public spaces, including in bodies of water, are also used when no formal waste collection infrastructure is available. This aspect of the calculations may have resulted in an overestimation of emissions at the city level. Future research would benefit from direct measurements of the mass of plastic waste burned domestically to provide more accurate estimates of emissions that come from the domestic burning of plastic waste.

Despite these limitations, our research has taken the first steps towards providing emission estimates from domestic burning of plastic waste in Guatemala. Domestic burning of plastic waste results in significant emissions; lowering these emissions by improving waste collection, particularly in low-income countries and rural areas, will be important for improving air quality, mitigating climate change, and reducing adverse human health impacts.

## Author contributions

MB: formal analysis, visualization, writing – original draft. ES: conceptualization, formal analysis, funding acquisition, methodology, supervision, writing – review & editing. MH: conceptualization, investigation, methodology, writing – review & editing. AR: software. JM: writing – review & editing. LT: conceptualization, investigation, funding acquisition, methodology, writing – review & editing.

## Conflicts of interest

There are no conflicts to declare.

## Supplementary Material

EA-003-D2EA00082B-s001
