# Supplementary material for: Plastic waste generation and emissions from the domestic open burning of plastic waste in Guatemala
Source: Environ Sci Atmos. 2022 Nov 12;3(1):156–67. doi: 10.1039/d2ea00082b (PMC9850929; doi:10.1039/d2ea00082b)
Supplement: EA-003-D2EA00082B-s001 [file EA-003-D2EA00082B-s001.pdf]

Electronic Supplementary Material (ESI) for Environmental Science: Atmospheres.
This journal is © The Royal Society of Chemistry 2022

Supplementary Information

Table S1. Survey questions for estimating exposure to plastic burning in Xalapán

Xalapán, Jalapa, Guatemala Survey
Estimation of exposure to plastic burning in rural homes in Jalapa, Guatemala
Questionnaire for adults participating in community activities in Xalapán

ID ___________ Date m-d-y______________________

Sex	Female<br>Male
Age	_____Years
1. How many people live in your home?	Number
2. What kind of stove do you use most of the time for cooking? [Choose the primary stove]	Open fire, three stone fire<br>Poyetón<br>Improved stove with chimney<br>Gas<br>Electric
3. What other types of stoves do you use to cook? [Choose others other than the primary stove]	Open fire, three stones<br>Poyetón<br>Improved stove with chimney<br>Gas<br>Electric
4. What methods do you currently use to get rid of garbage at your home?	Burned<br>Buried<br>Other
5. Are you currently studying?	Yes No
5a. How many years of formal education have you completed?	___Years
5b. What is the highest level of education you have completed?	1 = Without formal education<br>2 = Primary, incomplete<br>3 = Primary, complete<br>4 = Secondary, incomplete<br>5 = Secondary, complete<br>6 = Vocational or technical school<br>7 = University
6. Do you currently work?	Yes No
6a. If yes, what is your occupation?	
7. Do you have your own cell phone?	Yes No

8. If yes, is it a smartphone?	Yes No
9. Do you have a television in your home?	Yes No
10. Do you have a radio in your home?	Yes No
11.Do you have a computer in your home?	Yes No
12. Do you have access to internet?	Yes No
13. Weight in kilograms of plastic waste generated in the 1st. week	XX.XX
14. Weight in kilograms of plastic waste generated in the 2nd. week	XX.XX
15. P Weight in kilograms of plastic waste generated in the 3rd. week	XX.XX
16. Weight in kilograms of plastic waste generated in the 4th. week	XX.XX

Table S2. Census-based mass distribution of plastic waste burned per capita per year in kilograms and rural population percentage in each department in Guatemala

	Department	Lower boundary estimate of plastic waste burned per capita per year (kg/capita/year (SD))	Upper boundary estimate of plastic waste burned per capita per year[a] (kg/capita/year)	Rural population rate[b] (%)	Indigenous Population Rate[b] (%)	Poverty rate[c] (%)
01	Guatemala	1.26 (±0.594)	3.02	8.8	13.8	33.3
02	El Progreso	5.61 (±2.65)	13.49	48.2	1.7	53.2
03	Sacatepéquez	0.950 (±0.449)	2.29	11.5	40.5	41.1
04	Chimaltenango	4.08 (±1.93)	9.82	45.9	78.5	66.1
05	Escuintla	5.85 (±2.77)	14.1	38.8	5.4	52.9
06	Santa Rosa	6.65 (±3.15)	16.0	53.6	16.5	54.3
07	Sololá	3.47 (±1.64)	8.34	38.4	96.6	80.9
08	Totonicapán	6.15 (±2.91)	14.8	51.0	98.2	77.5
09	Quetzaltenango	4.48 (±2.12)	10.8	38.5	51.1	56.0
10	Suchitepéquez	6.37 (±3.01)	15.3	52.0	38.8	63.8
11	Retalhuleu	7.95 (±3.76)	19.1	42.7	15.3	56.1
12	San Marcos	7.02 (±3.32)	16.9	74.6	30.9	60.2
13	Huehuetenango	6.68 (±3.16)	16.1	72.0	65.2	73.8
14	Quiché	6.98 (±3.30)	16.8	67.7	89.4	74.7
15	Baja Verapaz	7.72 (±3.65)	18.6	59.8	60.3	66.3
16	Alta Verapaz	8.33 (±3.94)	20.0	68.7	93.2	83.1
17	Petén	9.65 (±4.56)	23.2	59.5	30.5	60.8
18	Izabal	7.19 (±3.40)	17.3	59.0	29.3	59.9
19	Zacapa	5.64 (±2.67)	13.6	55.8	2.4	55.9
20	Chiquimula	6.09 (±2.88)	14.6	62.6	27.2	70.6
21	Jalapa	6.04 (±2.86)	14.5	37.0	39.4	67.2
22	Jutiapa	7.44 (±3.52)	17.9	48.9	20.7	62.7

[a] Calculated in this study
[b] Calculated from the Guatemala Census
[c] Source: Instituto Nacional de Estadística Guatemala

Table S3. Plastic waste generated and theoretically burned per capita in Xalapán, Jalapa based on data collected in this study by the Xalapán, Jalapa, Guatemala Survey

	Xalapán, Jalapa (SD)
Total number of people in all households participating in this study	294
Mass of plastic waste generated per person per day (kg/capita/day)	3.34×10^{-2} ($\pm1.58\times10^{-2}$)
Mass of plastic waste generated per person per year (kg/capita/year)	12.2 (±5.8)
Percent of households that burn plastic (%)	84
Percent of the mass of plastic that would have been burned (%)	79.8
Mass of plastic waste theoretically burned per person per day (kg/capita/day)	2.66×10^{-2} ($\pm1.32\times10^{-2}$)
Mass of plastic waste theoretically burned per person per year (kg/capita/year)	9.72 (±4.81)

Table S4. Significance test results for relationship between the log transformed mass of waste generated and education level; cellphone, radio and color television ownership; and internet access.

Testing for relationship between the mass of waste generated each week:	Log ratio of average plastic waste generated	p-value
Week 1 and Week 2	0.110	0.07
Week 1 and Week 3	0.205	0.001
Week 1 and Week 4	0.162	0.007
Week 2 and Week 3	9.47×10^{-2}	0.16
Week 2 and Week 4	5.19×10^{-2}	0.42
Week 3 and Week 4	-4.28×10^{-2}	0.51

Table S5. Plastic Waste generated and burned per capita in Jutiapa; Guatemala City; and the country of Guatemala

	Jutiapa	Guatemala City	Guatemala
Total Per Capita Waste Generation[a] (kg/capita/day)	0.357	0.453	0.465
Percent of waste generated that is plastic[a] (%)	11.8	17.0	17.3
Mass of plastic waste generated per person per day[b] (kg/capita/day)	4.21×10^{-2}	7.70×10^{-2}	8.04×10^{-2}
Mass of plastic waste generated per person per year[b] (kg/capita/year)	15.4	28.1	29.3
Percent of households that burn plastic[b] (%)	61.0	10.3	42.8
Percent of the mass of plastic burned* (%)	87.5	28.0	NA
Mass of plastic waste burned per person per day[b] (kg/capita/day)	3.69×10^{-2}	2.16×10^{-2}	NA
Mass of plastic waste burned per person per year[b] (kg/capita/year)	13.5	7.87	NA

* Based on World Bank data on the percent mass of waste collected. The percent of waste not collected is assumed to have been burned for these calculations.

[a] Source: World Bank - What a Waste

[b] Calculated in this study

Table S6. Lower boundary annual per capita plastic waste burning emissions estimates using the departmental mass distribution from the La Fuente, Jalapa study-based

	Species	Jalapa		Jutiapa		Guatemala	
		Emissions estimate (kg/capita/ year)	SD	Emissions estimate (kg/capita/ year)	SD	Emissions estimate (kg/capita/ year)	SD
1	$PM_{2.5}$	0.505	0.254	0.623	0.313	0.105	5.25×10^{-2}
2	BC	6.23×10^{-2}	3.03×10^{-2}	7.67×10^{-2}	3.73×10^{-2}	1.30×10^{-2}	6.26×10^{-3}
3	OC	0.301	0.149	0.371	0.184	6.28×10^{-2}	3.09×10^{-2}
4	Ammonium	2.12×10^{-3}	1.78×10^{-3}	2.61×10^{-3}	2.19×10^{-3}	4.41×10^{-4}	3.69×10^{-4}
5	Chloride	5.13×10^{-3}	2.51×10^{-3}	6.33×10^{-3}	3.08×10^{-3}	1.07×10^{-3}	5.18×10^{-4}
6	Nitrate	2.60×10^{-3}	1.51×10^{-3}	3.20×10^{-3}	1.85×10^{-3}	5.41×10^{-4}	3.12×10^{-4}
7	Sulfate	1.03×10^{-3}	2.19×10^{-3}	1.27×10^{-3}	2.70×10^{-3}	2.14×10^{-4}	4.56×10^{-4}
8	Sb	3.75×10^{-6}	2.11×10^{-5}	4.62×10^{-6}	2.60×10^{-5}	7.82×10^{-7}	4.40×10^{-6}
9	Pb	8.20×10^{-5}	4.28×10^{-5}	1.01×10^{-4}	5.26×10^{-5}	1.71×10^{-5}	8.84×10^{-6}
10	Phenanthrene	3.19×10^{-5}	1.70×10^{-5}	3.94×10^{-5}	2.09×10^{-5}	6.66×10^{-6}	3.52×10^{-6}
11	Anthracene	4.71×10^{-6}	2.53×10^{-6}	5.81×10^{-6}	3.12×10^{-6}	9.83×10^{-7}	5.25×10^{-7}
12	Fluoranthene	7.58×10^{-5}	4.03×10^{-5}	9.34×10^{-5}	4.96×10^{-5}	1.58×10^{-5}	8.34×10^{-6}
13	Pyrene	8.27×10^{-5}	4.40×10^{-5}	1.02×10^{-4}	5.40×10^{-5}	1.72×10^{-5}	9.08×10^{-6}
14	Benzo(ghi)-fluoranthene	1.09×10^{-4}	5.81×10^{-5}	1.35×10^{-4}	7.14×10^{-5}	2.28×10^{-5}	1.20×10^{-5}
15	Cyclopenta-(cd)pyrene	4.90×10^{-5}	2.61×10^{-5}	6.04×10^{-5}	3.21×10^{-5}	1.02×10^{-5}	5.39×10^{-6}
16	Benz(a)-anthracene	6.30×10^{-5}	3.35×10^{-5}	7.77×10^{-5}	4.13×10^{-5}	1.31×10^{-5}	6.94×10^{-6}
17	Chrysene	9.19×10^{-5}	4.89×10^{-5}	1.13×10^{-4}	6.02×10^{-5}	1.92×10^{-5}	1.01×10^{-5}
18	Retene	1.71×10^{-5}	9.23×10^{-6}	2.11×10^{-5}	1.13×10^{-5}	3.56×10^{-6}	1.91×10^{-6}
19	Benzo(b)-fluoranthene	9.55×10^{-5}	5.08×10^{-5}	1.18×10^{-4}	6.25×10^{-5}	1.99×10^{-5}	1.05×10^{-5}
20	Benzo(k)-fluoranthene	3.99×10^{-5}	2.12×10^{-5}	4.91×10^{-5}	2.61×10^{-5}	8.31×10^{-6}	4.39×10^{-6}
21	Benzo(j)-fluoranthene	3.66×10^{-5}	1.95×10^{-5}	4.52×10^{-5}	2.40×10^{-5}	7.64×10^{-6}	4.04×10^{-6}
22	Benzo(e)-pyrene	3.26×10^{-5}	1.74×10^{-5}	4.02×10^{-5}	2.13×10^{-5}	6.80×10^{-6}	3.59×10^{-6}
23	Benzo(a)-pyrene	3.12×10^{-5}	1.66×10^{-5}	3.85×10^{-5}	2.04×10^{-5}	6.51×10^{-6}	3.44×10^{-6}
24	Perylene	9.54×10^{-6}	5.09×10^{-6}	1.18×10^{-5}	6.26×10^{-6}	1.99×10^{-6}	1.05×10^{-6}
25	Indeno(1,2,3-cd)pyrene	5.02×10^{-5}	2.67×10^{-5}	6.18×10^{-5}	3.28×10^{-5}	1.05×10^{-5}	5.52×10^{-6}
26	Benzo(GHI)-perylene	4.91×10^{-5}	2.62×10^{-5}	6.06×10^{-5}	3.22×10^{-5}	1.02×10^{-5}	5.41×10^{-6}
27	Dibenz(ah)-anthracene	1.66×10^{-5}	8.94×10^{-6}	2.05×10^{-5}	1.10×10^{-5}	3.46×10^{-6}	1.84×10^{-6}

28	Picene	2.88×10^{-5}	1.54×10^{-5}	3.55×10^{-5}	1.89×10^{-5}	6.01×10^{-6}	3.17×10^{-6}
29	Triphenyl-benzene	3.32×10^{-6}	1.83×10^{-6}	4.09×10^{-6}	2.25×10^{-6}	6.93×10^{-7}	3.79×10^{-7}
30	17a(H)-21b(H)-Hopane	5.92×10^{-6}	3.57×10^{-6}	7.29×10^{-6}	4.39×10^{-6}	1.23×10^{-6}	7.38×10^{-7}
31	Pristane	7.71×10^{-5}	5.87×10^{-5}	9.51×10^{-5}	7.24×10^{-5}	1.61×10^{-5}	1.22×10^{-5}
32	Norpristane	4.94×10^{-5}	3.03×10^{-5}	6.08×10^{-5}	3.72×10^{-5}	1.03×10^{-5}	6.26×10^{-6}
33	Phytane	1.04×10^{-5}	2.20×10^{-5}	1.28×10^{-5}	2.71×10^{-5}	2.17×10^{-6}	4.57×10^{-6}
34	Squalane	2.81×10^{-5}	4.34×10^{-5}	3.47×10^{-5}	5.34×10^{-5}	5.87×10^{-6}	9.02×10^{-6}
35	Octadecane	4.95×10^{-5}	2.94×10^{-5}	6.10×10^{-5}	3.62×10^{-5}	1.03×10^{-5}	6.08×10^{-6}
36	Nonadecane	7.82×10^{-5}	5.73×10^{-5}	9.65×10^{-5}	7.07×10^{-5}	1.63×10^{-5}	1.19×10^{-5}
37	Eicosane	2.06×10^{-4}	1.23×10^{-4}	2.54×10^{-4}	1.51×10^{-4}	4.29×10^{-5}	2.55×10^{-5}
38	Heneicosane	1.61×10^{-4}	8.63×10^{-5}	1.98×10^{-4}	1.06×10^{-4}	3.36×10^{-5}	1.78×10^{-5}
39	Docosane	2.58×10^{-4}	1.96×10^{-4}	3.17×10^{-4}	2.41×10^{-4}	5.37×10^{-5}	4.05×10^{-5}
40	Tricosane	2.22×10^{-4}	1.37×10^{-4}	2.74×10^{-4}	1.69×10^{-4}	4.63×10^{-5}	2.84×10^{-5}
41	Tetracosane	4.37×10^{-4}	2.65×10^{-4}	5.38×10^{-4}	3.26×10^{-4}	9.11×10^{-5}	5.48×10^{-5}
42	Pentacosane	1.42×10^{-4}	1.82×10^{-4}	1.75×10^{-4}	2.24×10^{-4}	2.95×10^{-5}	3.79×10^{-5}
43	Hexacosane	2.11×10^{-4}	2.20×10^{-4}	2.59×10^{-4}	2.71×10^{-4}	4.39×10^{-5}	4.57×10^{-5}
44	Heptacosane	1.52×10^{-4}	2.14×10^{-4}	1.87×10^{-4}	2.64×10^{-4}	3.16×10^{-5}	4.45×10^{-5}
45	Octacosane	4.00×10^{-4}	2.56×10^{-4}	4.93×10^{-4}	3.14×10^{-4}	8.35×10^{-5}	5.30×10^{-5}
46	Nonacosane	2.19×10^{-4}	1.77×10^{-4}	2.70×10^{-4}	2.19×10^{-4}	4.57×10^{-5}	3.69×10^{-5}
47	Triacontane	4.03×10^{-4}	2.47×10^{-4}	4.96×10^{-4}	3.04×10^{-4}	8.40×10^{-5}	5.12×10^{-5}
48	Hentria-contane	2.32×10^{-4}	1.57×10^{-4}	2.86×10^{-4}	1.93×10^{-4}	4.84×10^{-5}	3.25×10^{-5}
49	Dotria-contane	2.83×10^{-4}	1.66×10^{-4}	3.48×10^{-4}	2.04×10^{-4}	5.89×10^{-5}	3.43×10^{-5}
50	Tritria-contane	2.52×10^{-4}	1.48×10^{-4}	3.10×10^{-4}	1.82×10^{-4}	5.25×10^{-5}	3.06×10^{-5}
51	Tetratria-contane	3.27×10^{-4}	1.77×10^{-4}	4.03×10^{-4}	2.18×10^{-4}	6.82×10^{-5}	3.67×10^{-5}
52	Pentatria-contane	1.88×10^{-4}	1.05×10^{-4}	2.32×10^{-4}	1.29×10^{-4}	3.92×10^{-5}	2.17×10^{-5}
53	Levo-glucosan	4.20×10^{-3}	2.24×10^{-3}	5.17×10^{-3}	2.75×10^{-3}	8.75×10^{-4}	4.62×10^{-4}
54	Stigmasterol	2.78×10^{-5}	1.69×10^{-5}	3.42×10^{-5}	2.08×10^{-5}	5.79×10^{-6}	3.50×10^{-6}
55	b-Sitosterol	2.08×10^{-4}	1.21×10^{-4}	2.56×10^{-4}	1.49×10^{-4}	4.34×10^{-5}	2.51×10^{-5}
56	Campesterol	4.21×10^{-5}	2.32×10^{-5}	5.19×10^{-5}	2.86×10^{-5}	8.78×10^{-6}	4.80×10^{-6}
57	CO	0.238	0.243	0.293	0.300	4.96×10^{-2}	5.06×10^{-2}
58	CH_4	8.22×10^{-3}	7.50×10^{-3}	1.01×10^{-2}	9.24×10^{-3}	1.71×10^{-3}	1.55×10^{-3}
59	C_2H_2	7.65×10^{-3}	9.84×10^{-3}	9.42×10^{-3}	1.21×10^{-2}	1.59×10^{-3}	2.04×10^{-3}
60	C_2H_4	2.97×10^{-2}	4.42×10^{-2}	3.67×10^{-2}	5.45×10^{-2}	6.21×10^{-3}	9.22×10^{-3}
61	C_3H_6	1.11×10^{-2}	1.68×10^{-2}	1.37×10^{-2}	2.07×10^{-2}	2.32×10^{-3}	3.49×10^{-3}
62	CO_2	15.6	7.47	19.2	9.19	3.25	1.54

Table S7. Upper boundary annual per capita plastic waste burning emissions estimates using the departmental mass distribution from Guatemala Census tract

	Species	Jalapa		Jutiapa		Guatemala	
		Emissions estimate (kg/capita/year)	(SD)	Emissions estimate (kg/capita/year)	(SD)	Emissions estimate (kg/capita/year)	(SD)
1	$PM_{2.5}$	1.21	0.184	1.50	0.227	0.253	$3.85×10^{-2}$
2	BC	0.150	$1.51×10^{-2}$	0.185	$1.86×10^{-2}$	$3.11×10^{-2}$	$3.14×10^{-3}$
3	OC	0.723	$9.58×10^{-2}$	0.893	0.118	0.151	$2.00×10^{-2}$
4	Ammonium	$5.08×10^{-3}$	$3.19×10^{-3}$	$6.28×10^{-3}$	$3.94×10^{-3}$	$1.06×10^{-3}$	$6.65×10^{-4}$
5	Chloride	$1.23×10^{-2}$	$1.31×10^{-3}$	$1.52×10^{-2}$	$1.61×10^{-3}$	$2.57×10^{-3}$	$2.72×10^{-4}$
6	Nitrate	$6.24×10^{-3}$	$1.88×10^{-3}$	$7.70×10^{-3}$	$2.33×10^{-3}$	$1.30×10^{-3}$	$3.93×10^{-4}$
7	Sulfate	$2.47×10^{-3}$	$4.64×10^{-3}$	$3.05×10^{-3}$	$5.73×10^{-3}$	$5.15×10^{-4}$	$9.66×10^{-4}$
8	Sb	$8.94×10^{-6}$	$4.57×10^{-5}$	$1.10×10^{-5}$	$5.64×10^{-5}$	$1.87×10^{-6}$	$9.52×10^{-6}$
9	Pb	$1.97×10^{-4}$	$3.87×10^{-5}$	$2.43×10^{-4}$	$4.77×10^{-5}$	$4.10×10^{-5}$	$8.06×10^{-6}$
10	Phenanthrene	$7.67×10^{-5}$	$1.68×10^{-5}$	$9.47×10^{-5}$	$2.08×10^{-5}$	$1.60×10^{-5}$	$3.50×10^{-6}$
11	Anthracene	$1.13×10^{-5}$	$2.61×10^{-6}$	$1.40×10^{-5}$	$3.22×10^{-6}$	$2.36×10^{-6}$	$5.44×10^{-7}$
12	Fluoranthene	$1.82×10^{-4}$	$3.98×10^{-5}$	$2.25×10^{-4}$	$4.91×10^{-5}$	$3.79×10^{-5}$	$8.28×10^{-6}$
13	Pyrene	$1.98×10^{-4}$	$4.32×10^{-5}$	$2.45×10^{-4}$	$5.33×10^{-5}$	$4.13×10^{-5}$	$9.01×10^{-6}$
14	Benzo(ghi)-fluoranthene	$2.62×10^{-4}$	$5.71×10^{-5}$	$3.24×10^{-4}$	$7.05×10^{-5}$	$5.46×10^{-5}$	$1.19×10^{-5}$
15	Cyclopenta-(cd)pyrene	$1.18×10^{-4}$	$2.57×10^{-5}$	$1.45×10^{-4}$	$3.17×10^{-5}$	$2.45×10^{-5}$	$5.35×10^{-6}$
16	Benz(a)-anthracene	$1.51×10^{-4}$	$3.31×10^{-5}$	$1.87×10^{-4}$	$4.08×10^{-5}$	$3.16×10^{-5}$	$6.89×10^{-6}$
17	Chrysene	$2.21×10^{-4}$	$4.82×10^{-5}$	$2.73×10^{-4}$	$5.95×10^{-5}$	$4.60×10^{-5}$	$1.00×10^{-5}$
18	Retene	$4.10×10^{-5}$	$9.55×10^{-6}$	$5.07×10^{-5}$	$1.18×10^{-5}$	$8.55×10^{-6}$	$1.99×10^{-6}$
19	Benzo(b)-fluoranthene	$2.29×10^{-4}$	$5.00×10^{-5}$	$2.83×10^{-4}$	$6.17×10^{-5}$	$4.78×10^{-5}$	$1.04×10^{-5}$
20	Benzo(k)-fluoranthene	$9.57×10^{-5}$	$2.10×10^{-5}$	$1.18×10^{-4}$	$2.60×10^{-5}$	$1.99×10^{-5}$	$4.39×10^{-6}$
21	Benzo(j)-fluoranthene	$8.81×10^{-5}$	$1.93×10^{-5}$	$1.09×10^{-4}$	$2.38×10^{-5}$	$1.83×10^{-5}$	$4.02×10^{-6}$
22	Benzo(e)-pyrene	$7.83×10^{-5}$	$1.71×10^{-5}$	$9.67×10^{-5}$	$2.11×10^{-5}$	$1.63×10^{-5}$	$3.57×10^{-6}$
23	Benzo(a)-pyrene	$7.50×10^{-5}$	$1.64×10^{-5}$	$9.26×10^{-5}$	$2.02×10^{-5}$	$1.56×10^{-5}$	$3.42×10^{-6}$
24	Perylene	$2.29×10^{-5}$	$5.08×10^{-6}$	$2.83×10^{-5}$	$6.27×10^{-6}$	$4.78×10^{-6}$	$1.06×10^{-6}$
25	Indeno(1,2,3-cd)pyrene	$1.21×10^{-4}$	$2.63×10^{-5}$	$1.49×10^{-4}$	$3.24×10^{-5}$	$2.51×10^{-5}$	$5.46×10^{-6}$
26	Benzo(GHI)-perylene	$1.18×10^{-4}$	$2.58×10^{-5}$	$1.46×10^{-4}$	$3.18×10^{-5}$	$2.46×10^{-5}$	$5.38×10^{-6}$
27	Dibenz(ah)-anthracene	$3.99×10^{-5}$	$9.13×10^{-6}$	$4.92×10^{-5}$	$1.13×10^{-5}$	$8.31×10^{-6}$	$1.90×10^{-6}$

28	Picene	$6.91{\times}10^{-5}$	$1.52{\times}10^{-5}$	$8.53{\times}10^{-5}$	$1.88{\times}10^{-5}$	$1.44{\times}10^{-5}$	$3.17{\times}10^{-6}$
29	Triphenyl-benzene	$7.98{\times}10^{-6}$	$2.03{\times}10^{-6}$	$9.85{\times}10^{-6}$	$2.50{\times}10^{-6}$	$1.66{\times}10^{-6}$	$4.23{\times}10^{-7}$
30	17a(H)-21b(H)-Hopane	$1.42{\times}10^{-5}$	$4.79{\times}10^{-6}$	$1.75{\times}10^{-5}$	$5.91{\times}10^{-6}$	$2.96{\times}10^{-6}$	$9.97{\times}10^{-7}$
31	Pristane	$1.85{\times}10^{-4}$	$9.99{\times}10^{-5}$	$2.29{\times}10^{-4}$	$1.23{\times}10^{-4}$	$3.86{\times}10^{-5}$	$2.08{\times}10^{-5}$
32	Norpristane	$1.18{\times}10^{-4}$	$4.17{\times}10^{-5}$	$1.46{\times}10^{-4}$	$5.14{\times}10^{-5}$	$2.47{\times}10^{-5}$	$8.67{\times}10^{-6}$
33	Phytane	$2.50{\times}10^{-5}$	$4.66{\times}10^{-5}$	$3.08{\times}10^{-5}$	$5.75{\times}10^{-5}$	$5.21{\times}10^{-6}$	$9.69{\times}10^{-6}$
34	Squalane	$6.74{\times}10^{-5}$	$8.95{\times}10^{-5}$	$8.32{\times}10^{-5}$	$1.11{\times}10^{-4}$	$1.41{\times}10^{-5}$	$1.86{\times}10^{-5}$
35	Octadecane	$1.19{\times}10^{-4}$	$3.85{\times}10^{-5}$	$1.47{\times}10^{-4}$	$4.75{\times}10^{-5}$	$2.47{\times}10^{-5}$	$8.02{\times}10^{-6}$
36	Nonadecane	$1.88{\times}10^{-4}$	$9.48{\times}10^{-5}$	$2.32{\times}10^{-4}$	$1.17{\times}10^{-4}$	$3.91{\times}10^{-5}$	$1.97{\times}10^{-5}$
37	Eicosane	$4.94{\times}10^{-4}$	$1.63{\times}10^{-4}$	$6.10{\times}10^{-4}$	$2.01{\times}10^{-4}$	$1.03{\times}10^{-4}$	$3.39{\times}10^{-5}$
38	Heneicosane	$3.86{\times}10^{-4}$	$8.76{\times}10^{-5}$	$4.77{\times}10^{-4}$	$1.08{\times}10^{-4}$	$8.05{\times}10^{-5}$	$1.83{\times}10^{-5}$
39	Docosane	$6.19{\times}10^{-4}$	$3.31{\times}10^{-4}$	$7.64{\times}10^{-4}$	$4.09{\times}10^{-4}$	$1.29{\times}10^{-4}$	$6.91{\times}10^{-5}$
40	Tricosane	$5.33{\times}10^{-4}$	$1.91{\times}10^{-4}$	$6.58{\times}10^{-4}$	$2.35{\times}10^{-4}$	$1.11{\times}10^{-4}$	$3.97{\times}10^{-5}$
41	Tetracosane	$1.05{\times}10^{-3}$	$3.59{\times}10^{-4}$	$1.29{\times}10^{-3}$	$4.43{\times}10^{-4}$	$2.18{\times}10^{-4}$	$7.48{\times}10^{-5}$
42	Pentacosane	$3.40{\times}10^{-4}$	$3.68{\times}10^{-4}$	$4.20{\times}10^{-4}$	$4.54{\times}10^{-4}$	$7.09{\times}10^{-5}$	$7.67{\times}10^{-5}$
43	Hexacosane	$5.06{\times}10^{-4}$	$4.26{\times}10^{-4}$	$6.25{\times}10^{-4}$	$5.26{\times}10^{-4}$	$1.05{\times}10^{-4}$	$8.86{\times}10^{-5}$
44	Heptacosane	$3.64{\times}10^{-4}$	$4.37{\times}10^{-4}$	$4.49{\times}10^{-4}$	$5.40{\times}10^{-4}$	$7.58{\times}10^{-5}$	$9.10{\times}10^{-5}$
45	Octacosane	$9.60{\times}10^{-4}$	$3.72{\times}10^{-4}$	$1.19{\times}10^{-3}$	$4.59{\times}10^{-4}$	$2.00{\times}10^{-4}$	$7.75{\times}10^{-5}$
46	Nonacosane	$5.27{\times}10^{-4}$	$3.12{\times}10^{-4}$	$6.50{\times}10^{-4}$	$3.85{\times}10^{-4}$	$1.10{\times}10^{-4}$	$6.51{\times}10^{-5}$
47	Triacontane	$9.66{\times}10^{-4}$	$3.40{\times}10^{-4}$	$1.19{\times}10^{-3}$	$4.20{\times}10^{-4}$	$2.01{\times}10^{-4}$	$7.09{\times}10^{-5}$
48	Hentria-contane	$5.57{\times}10^{-4}$	$2.42{\times}10^{-4}$	$6.87{\times}10^{-4}$	$2.99{\times}10^{-4}$	$1.16{\times}10^{-4}$	$5.05{\times}10^{-5}$
49	Dotria-contane	$6.78{\times}10^{-4}$	$2.12{\times}10^{-4}$	$8.38{\times}10^{-4}$	$2.61{\times}10^{-4}$	$1.41{\times}10^{-4}$	$4.40{\times}10^{-5}$
50	Tritria-contane	$6.05{\times}10^{-4}$	$1.90{\times}10^{-4}$	$7.46{\times}10^{-4}$	$2.35{\times}10^{-4}$	$1.26{\times}10^{-4}$	$3.96{\times}10^{-5}$
51	Tetratria-contane	$7.84{\times}10^{-4}$	$1.87{\times}10^{-4}$	$9.68{\times}10^{-4}$	$2.31{\times}10^{-4}$	$1.63{\times}10^{-4}$	$3.90{\times}10^{-5}$
52	Pentatria-contane	$4.52{\times}10^{-4}$	$1.19{\times}10^{-4}$	$5.58{\times}10^{-4}$	$1.47{\times}10^{-4}$	$9.41{\times}10^{-5}$	$2.47{\times}10^{-5}$
53	Levo-glucosan	$1.01{\times}10^{-2}$	$2.22{\times}10^{-3}$	$1.24{\times}10^{-2}$	$2.74{\times}10^{-3}$	$2.10{\times}10^{-3}$	$4.63{\times}10^{-4}$
54	Stigmasterol	$6.67{\times}10^{-5}$	$2.31{\times}10^{-5}$	$8.23{\times}10^{-5}$	$2.85{\times}10^{-5}$	$1.39{\times}10^{-5}$	$4.81{\times}10^{-6}$
55	b-Sitosterol	$5.00{\times}10^{-4}$	$1.53{\times}10^{-4}$	$6.17{\times}10^{-4}$	$1.89{\times}10^{-4}$	$1.04{\times}10^{-4}$	$3.18{\times}10^{-5}$
56	Campesterol	$1.01{\times}10^{-4}$	$2.58{\times}10^{-5}$	$1.25{\times}10^{-4}$	$3.19{\times}10^{-5}$	$2.11{\times}10^{-5}$	$5.38{\times}10^{-6}$
57	CO	0.571	0.468	0.705	0.577	0.119	$9.73{\times}10^{-2}$
58	CH_4	$1.98{\times}10^{-2}$	$1.39{\times}10^{-2}$v	$2.44{\times}10^{-2}$	$1.72{\times}10^{-2}$	$4.11{\times}10^{-3}$	$2.90{\times}10^{-3}$
59	C_2H_2	$1.83{\times}10^{-2}$	$1.98{\times}10^{-2}$	$2.26{\times}10^{-2}$	$2.45{\times}10^{-2}$	$3.82{\times}10^{-3}$	$4.13{\times}10^{-3}$
60	C_2H_4	$7.14{\times}10^{-2}$	$9.10{\times}10^{-2}$	$8.82{\times}10^{-2}$	0.112	$1.49{\times}10^{-2}$	$1.90{\times}10^{-2}$
61	C_3H_6	$2.67{\times}10^{-2}$	$3.47{\times}10^{-2}$	$3.30{\times}10^{-2}$	$4.28{\times}10^{-2}$	$5.56{\times}10^{-3}$	$7.22{\times}10^{-3}$
62	CO_2	37.5	2.28	46.3	2.81	7.81	0.474

Table S8. Lower boundary estimated annual emissions from plastic waste burning for $PM_{2.5}$, EC, OC, CO, and CO_2 for each department in Guatemala using La Fuente, Jalapa study

	Department	$PM_{2.5}$		BC		OC	
		Emissions estimate (kg/year)	SD	Emissions estimate (kg/year)	SD	Emissions estimate (kg/year)	SD
01	Guatemala	3.18×10^5	1.58×10^5	3.92×10^4	1.89×10^4	1.89×10^5	9.30×10^4
02	El Progreso	8.30×10^4	4.16×10^4	1.02×10^4	4.96×10^3	4.95×10^4	2.44×10^4
03	Sacatepéquez	2.63×10^4	1.32×10^4	3.24×10^3	1.57×10^3	1.57×10^4	7.75×10^3
04	Chimaltenango	2.10×10^5	1.05×10^5	2.59×10^4	1.26×10^4	1.25×10^5	6.20×10^4
05	Escuintla	3.59×10^5	1.80×10^5	4.42×10^4	2.15×10^4	2.14×10^5	1.06×10^5
06	Santa Rosa	2.21×10^5	1.11×10^5	2.72×10^4	1.32×10^4	1.31×10^5	6.52×10^4
07	Sololá	1.22×10^5	6.14×10^4	1.51×10^4	7.32×10^3	7.29×10^4	3.61×10^4
08	Totonicapán	2.15×10^5	1.08×10^5	2.65×10^4	1.29×10^4	1.28×10^5	6.36×10^4
09	Quetzaltenango	2.99×10^5	1.50×10^5	3.69×10^4	1.80×10^4	1.78×10^5	8.84×10^4
10	Suchitepéquez	2.96×10^5	1.48×10^5	3.64×10^4	1.77×10^4	1.76×10^5	8.73×10^4
11	Retalhuleu	2.17×10^5	1.09×10^5	2.68×10^4	1.30×10^4	1.30×10^5	6.42×10^4
12	San Marcos	6.07×10^5	3.04×10^5	7.48×10^4	3.63×10^4	3.62×10^5	1.79×10^5
13	Huehuetenango	6.54×10^5	3.28×10^5	8.06×10^4	3.92×10^4	3.90×10^5	1.93×10^5
14	Quiche	5.55×10^5	2.78×10^5	6.83×10^4	3.32×10^4	3.30×10^5	1.63×10^5
15	Baja Verapaz	1.93×10^5	9.71×10^4	2.38×10^4	1.16×10^4	1.15×10^5	5.70×10^4
16	Alta Verapaz	8.46×10^5	4.25×10^5	1.04×10^5	5.08×10^4	5.04×10^5	2.50×10^5
17	Petén	4.41×10^5	2.21×10^5	5.43×10^4	2.64×10^4	2.63×10^5	1.30×10^5
18	Izabal	2.46×10^5	1.23×10^5	3.03×10^4	1.47×10^4	1.47×10^5	7.25×10^4
19	Zacapa	1.16×10^5	5.82×10^4	1.43×10^4	6.95×10^3	6.90×10^4	3.42×10^4
20	Chiquimula	2.11×10^5	1.06×10^5	2.60×10^4	1.27×10^4	1.26×10^5	6.24×10^4
21	Jalapa	1.73×10^5	8.72×10^4	2.14×10^4	1.04×10^4	1.03×10^5	5.12×10^4
22	Jutiapa	3.04×10^5	1.53×10^5	3.75×10^4	1.82×10^4	1.81×10^5	8.97×10^4
	Total	6.71×10^6	3.37×10^6	8.27×10^5	4.02×10^5	4.00×10^6	1.98×10^6

		CO		CO_2	
		Emissions (kg/yr)	SD	Emissions (kg/yr)	SD
01	Guatemala	1.50×10^5	1.52×10^5	9.82×10^6	4.65×10^6
02	El Progreso	3.91×10^4	3.98×10^4	2.56×10^6	1.22×10^6
03	Sacatepéquez	1.24×10^4	1.26×10^4	8.11×10^5	3.87×10^5
04	Chimaltenango	9.91×10^4	1.01×10^5	6.49×10^6	3.10×10^6
05	Escuintla	1.69×10^5	1.73×10^5	1.11×10^7	5.30×10^6
06	Santa Rosa	1.04×10^5	1.06×10^5	6.82×10^6	3.26×10^6
07	Sololá	5.76×10^4	5.88×10^4	3.78×10^6	1.80×10^6
08	Totonicapán	1.01×10^5	1.04×10^5	6.65×10^6	3.18×10^6
09	Quetzaltenango	1.41×10^5	1.44×10^5	9.25×10^6	4.42×10^6
10	Suchitepéquez	1.39×10^5	1.42×10^5	9.13×10^6	4.36×10^6
11	Retalhuleu	1.02×10^5	1.05×10^5	6.72×10^6	3.21×10^6
12	San Marcos	2.86×10^5	2.92×10^5	1.87×10^7	8.94×10^6

13	Huehuetenango	3.08×10^5	3.14×10^5	2.02×10^7	9.65×10^6
14	Quiché	2.61×10^5	2.66×10^5	1.71×10^7	8.17×10^6
15	Baja Verapaz	9.12×10^4	9.30×10^4	5.97×10^6	2.85×10^6
16	Alta Verapaz	3.99×10^5	4.07×10^5	2.61×10^7	1.25×10^7
17	Petén	2.08×10^5	2.12×10^5	1.36×10^7	6.49×10^6
18	Izabal	1.16×10^5	1.18×10^5	7.60×10^6	3.63×10^6
19	Zacapa	5.46×10^4	5.57×10^4	3.58×10^6	1.71×10^6
20	Chiquimula	9.96×10^4	1.02×10^5	6.53×10^6	3.12×10^6
21	Jalapa	8.16×10^4	8.33×10^4	5.35×10^6	2.56×10^6
22	Jutiapa	1.43×10^5	1.46×10^5	9.39×10^6	4.48×10^6
	Total	3.16×10^6	3.23×10^6	2.07×10^8	9.90×10^7

Table S9. Upper boundary estimated annual emissions from plastic waste burning for $PM_{2.5}$, EC, OC, CO, and CO_2 for each department in Guatemala using Guatemala Census data

	Department	$PM_{2.5}$		BC		OC	
		Emissions estimate (kg/year)	SD	Emissions estimate (kg/year)	SD	Emissions estimate(kg/year)	SD
01	Guatemala	7.62×10^{5}	1.16×10^{5}	9.39×10^{4}	9.48×10^{3}	4.54×10^{5}	6.02×10^{4}
02	El Progreso	2.00×10^{5}	3.04×10^{4}	2.46×10^{4}	2.48×10^{3}	1.19×10^{5}	1.58×10^{4}
03	Sacatepéquez	6.33×10^{4}	9.64×10^{3}	7.80×10^{3}	7.87×10^{2}	3.77×10^{4}	5.00×10^{3}
04	Chimaltenango	5.06×10^{5}	7.70×10^{4}	6.23×10^{4}	6.29×10^{3}	3.02×10^{5}	4.00×10^{4}
05	Escuintla	8.65×10^{5}	1.32×10^{5}	1.07×10^{5}	1.08×10^{4}	5.16×10^{5}	6.83×10^{4}
06	Santa Rosa	5.31×10^{5}	8.08×10^{4}	6.54×10^{4}	6.60×10^{3}	3.16×10^{5}	4.19×10^{4}
07	Sololá	2.94×10^{5}	4.48×10^{4}	3.62×10^{4}	3.66×10^{3}	1.75×10^{5}	2.32×10^{4}
08	Totonicapán	5.18×10^{5}	7.89×10^{4}	6.39×10^{4}	6.45×10^{3}	3.09×10^{5}	4.09×10^{4}
09	Quetzaltenango	7.22×10^{5}	1.10×10^{5}	8.90×10^{4}	8.98×10^{3}	4.30×10^{5}	5.70×10^{4}
10	Suchitepéquez	7.10×10^{5}	1.08×10^{5}	8.75×10^{4}	8.83×10^{3}	4.23×10^{5}	5.61×10^{4}
11	Retalhuleu	5.22×10^{5}	7.95×10^{4}	6.44×10^{4}	6.50×10^{3}	3.11×10^{5}	4.12×10^{4}
12	San Marcos	1.46×10^{6}	2.22×10^{5}	1.80×10^{5}	1.82×10^{4}	8.70×10^{5}	1.15×10^{5}
13	Huehuetenango	1.58×10^{6}	2.40×10^{5}	1.94×10^{5}	1.96×10^{4}	9.40×10^{5}	1.25×10^{5}
14	Quiche	1.33×10^{6}	2.03×10^{5}	1.64×10^{5}	1.66×10^{4}	7.95×10^{5}	1.05×10^{5}
15	Baja Verapaz	4.66×10^{5}	7.10×10^{4}	5.74×10^{4}	5.80×10^{3}	2.78×10^{5}	3.68×10^{4}
16	Alta Verapaz	2.03×10^{6}	3.10×10^{5}	2.51×10^{5}	2.53×10^{4}	1.21×10^{6}	1.61×10^{5}
17	Petén	1.06×10^{6}	1.61×10^{5}	1.30×10^{5}	1.32×10^{4}	6.31×10^{5}	8.36×10^{4}
18	Izabal	5.92×10^{5}	9.01×10^{4}	7.29×10^{4}	7.36×10^{3}	3.53×10^{5}	4.67×10^{4}
19	Zacapa	2.79×10^{5}	4.25×10^{4}	3.44×10^{4}	3.47×10^{3}	1.66×10^{5}	2.20×10^{4}
20	Chiquimula	5.07×10^{5}	7.72×10^{4}	6.25×10^{4}	6.31×10^{3}	3.02×10^{5}	4.00×10^{4}
21	Jalapa	4.16×10^{5}	6.33×10^{4}	5.13×10^{4}	5.17×10^{3}	2.48×10^{5}	3.29×10^{4}
22	Jutiapa	7.32×10^{5}	1.11×10^{5}	9.01×10^{4}	9.10×10^{3}	4.36×10^{5}	5.78×10^{4}
	Total	1.61×10^{7}	2.46×10^{6}	1.99×10^{6}	2.01×10^{5}	9.62×10^{6}	1.28×10^{6}

		CO		CO_2	
		Emissions (kg/year)	SD	Emissions (kg/year)	SD
01	Guatemala	3.59×10^{5}	2.93×10^{5}	2.35×10^{7}	1.43×10^{6}
02	El Progreso	9.40×10^{4}	7.68×10^{4}	6.16×10^{6}	3.75×10^{5}
03	Sacatepéquez	2.98×10^{4}	2.44×10^{4}	1.96×10^{6}	1.19×10^{5}
04	Chimaltenango	2.38×10^{5}	1.95×10^{5}	1.56×10^{7}	9.50×10^{5}
05	Escuintla	4.07×10^{5}	3.33×10^{5}	2.67×10^{7}	1.62×10^{6}
06	Santa Rosa	2.50×10^{5}	2.04×10^{5}	1.64×10^{7}	9.97×10^{5}
07	Sololá	1.39×10^{5}	1.13×10^{5}	9.09×10^{6}	5.52×10^{5}
08	Totonicapán	2.44×10^{5}	1.99×10^{5}	1.60×10^{7}	9.73×10^{5}
09	Quetzaltenango	3.40×10^{5}	2.78×10^{5}	2.23×10^{7}	1.36×10^{6}
10	Suchitepéquez	3.35×10^{5}	2.73×10^{5}	2.19×10^{7}	1.33×10^{6}
11	Retalhuleu	2.46×10^{5}	2.01×10^{5}	1.61×10^{7}	9.81×10^{5}
12	San Marcos	6.88×10^{5}	5.62×10^{5}	4.51×10^{7}	2.74×10^{6}

13	Huehuetenango	7.43×10^5	6.07×10^5	4.87×10^7	2.96×10^6
14	Quiché	6.29×10^5	5.13×10^5	4.12×10^7	2.51×10^6
15	Baja Verapaz	2.20×10^5	1.79×10^5	1.44×10^7	8.75×10^5
16	Alta Verapaz	9.58×10^5	7.82×10^5	6.28×10^7	3.82×10^6
17	Petén	4.99×10^5	4.08×10^5	3.27×10^7	1.99×10^6
18	Izabal	2.79×10^5	2.28×10^5	1.83×10^7	1.11×10^6
19	Zacapa	1.32×10^5	1.07×10^5	8.62×10^6	5.24×10^5
20	Chiquimula	2.39×10^5	1.95×10^5	1.57×10^7	9.52×10^5
21	Jalapa	1.96×10^5	1.60×10^5	1.28×10^7	7.81×10^5
22	Jutiapa	3.45×10^5	2.81×10^5	2.26×10^7	1.37×10^6
	Total	7.61×10^6	6.21×10^6	4.99×10^8	3.03×10^7

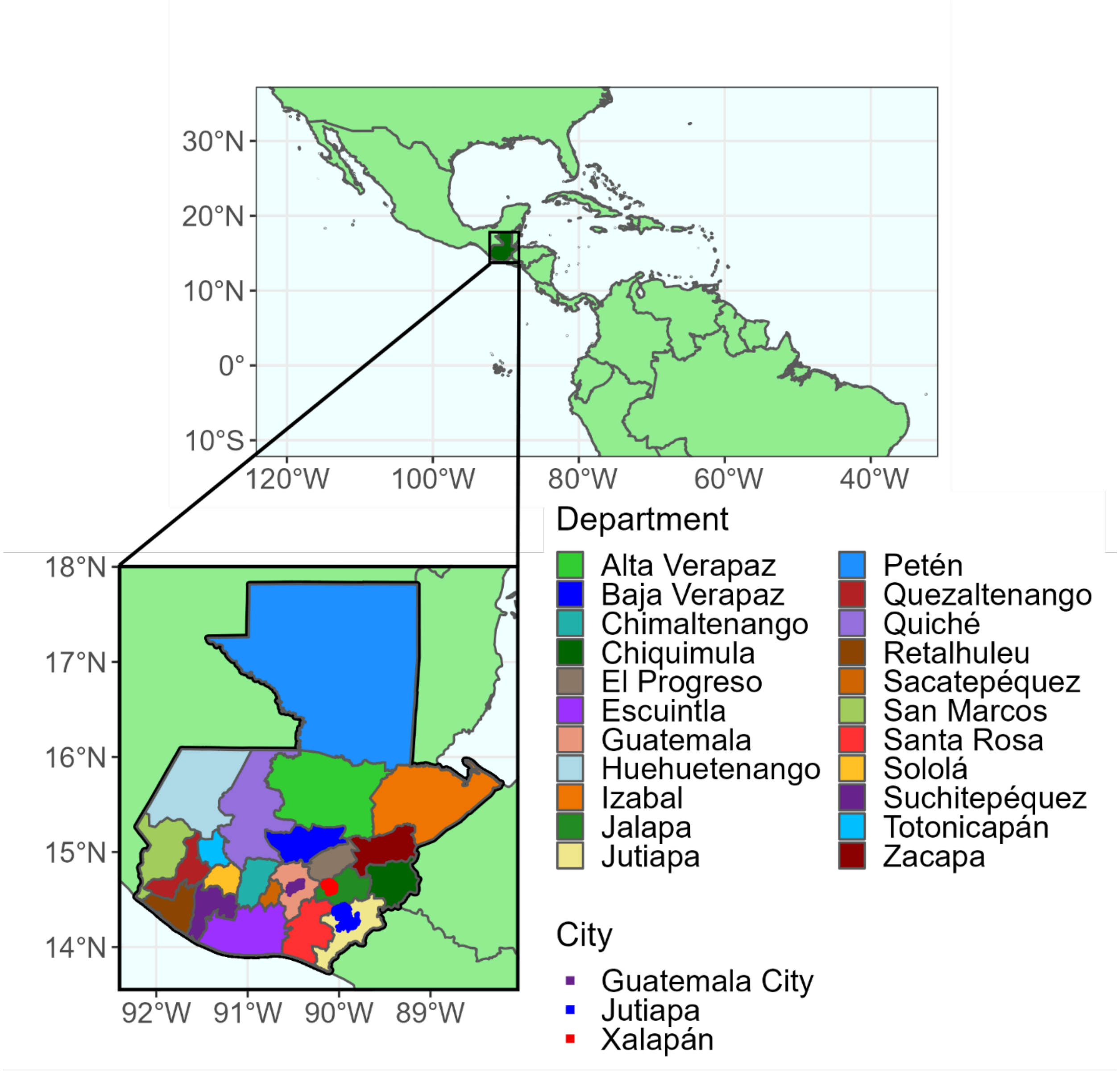

Figure S1. Map indicating location of Guatemala, Guatemala Departments, and the cities of Guatemala, Jutiapa, and Xalapán

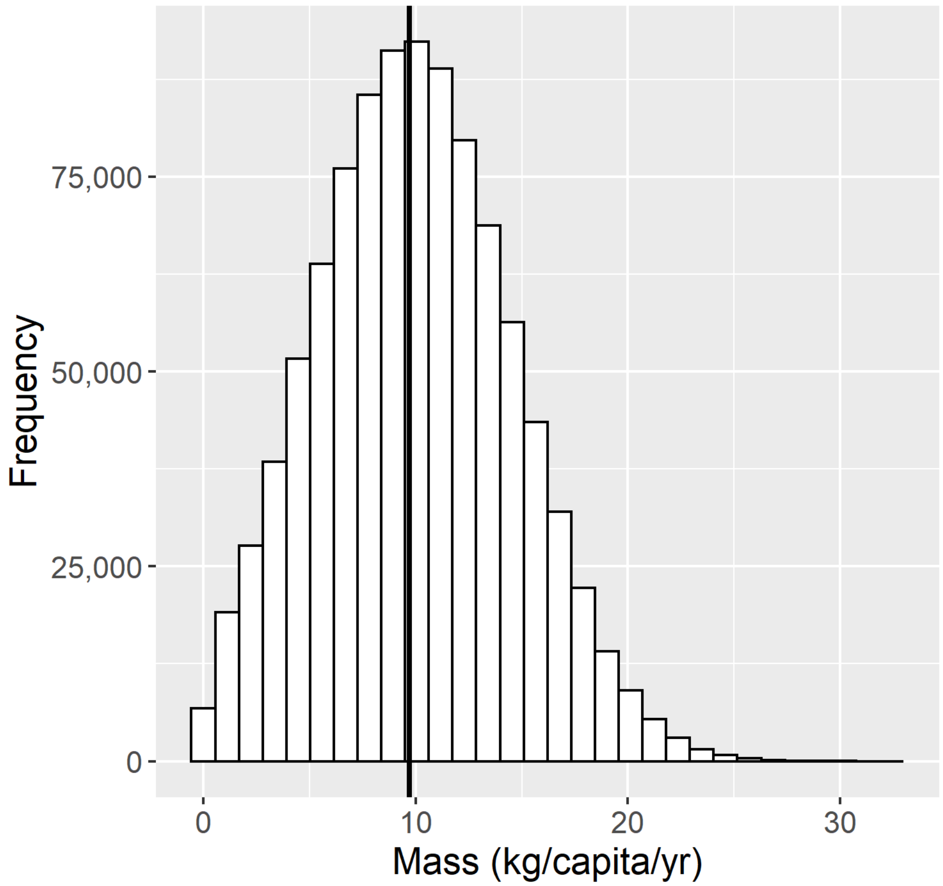

Figure S2. Distribution of Mass of Plastic Waste Theoretically Burned in Xalapán, Jalapa Based on Monte Carlo Samplings.
